# Working memory updating of emotional stimuli predicts emotional intelligence in females

**DOI:** 10.1038/s41598-020-77944-9

**Published:** 2020-11-30

**Authors:** Jarosław Orzechowski, Magdalena Śmieja, Karol Lewczuk, Edward Nęcka

**Affiliations:** 1 SWPS University of Social Sciences and Humanities, Warsaw, Poland; 2 Jagiellonian University, Kraków, Poland; 3 Cardinal Stefan Wyszyński University, Warsaw, Poland

**Keywords:** Psychology, Human behaviour

## Abstract

Preliminary evidence concerning emotional intelligence (EI) and working memory (WM) showed that the relationship between them is dependent on the emotional content (‘hot’ or ‘cool’) of tasks involving WM. In this paper, we continue investigating the relationship between EI and WM, focusing on a crucial function of WM, i.e., the efficacy of updating its content. WM updating shows substantial correlations with general fluid intelligence (gF) and seems to be a significant predictor of cognitive performance and achievement. We assume that if updating is important for a wide range of higher-order processes, updating emotional content in WM could be essential for emotionally intelligent behavior. To test this hypothesis, we constructed two parallel versions of a task that requires WM updating: one with neutral and the other with emotional stimuli. In addition, performance-based measures of both gF and EI were used in the research. Using the structural equation approach, we sought to demonstrate that gF is dependent on the efficiency of WM updating for both emotional and neutral stimuli, whereas EI might depend only on the updating efficacy in the emotional context. The results are discussed in terms of the domain specificity of EI and the domain generality of gF. The main constraint of the study is its limited sample size (n = 123 for intelligence measures, n = 69 for WM updating tasks). Moreover, the study was based on a female sample; thus, the conclusions can be extrapolated only to women.

## Introduction

Working memory capacity (WMC) is an important predictor of general fluid intelligence (gF), which in turn strongly correlates with Spearman’s^1^ general factor *g*^2^. The significance of working memory (WM) processes for human intelligence has been demonstrated in many studies^3–8^. The WMC-gF connection is usually interpreted in terms of the cognitive underpinnings of human intelligence: detailed and basic processes constituting WM are believed to account for the global trait called intelligence (see:^9^).

Both WM and gF are ‘general purpose’ mechanisms, meaning that they can be expressed in a variety of tasks and situations. For instance, WMC predicts not only gF but also reading skills^10,11^, verbal comprehension^12,13^, mathematical skills^14^, and problem-solving skills^9,15,16^. Additionally, gF has been defined as the domain-general ability “to reason, plan, solve problems, think abstractly, comprehend complex ideas, learn quickly and learn from experience”^17^, p. 13. This ‘general’ nature of both constructs implies that individual differences in gF and WMC, as well as the relationships between them, should not depend on the type of material processed (verbal vs. nonverbal, emotional vs. neutral). On the other hand, emotional intelligence (EI) seems much more dependent on the specificity of the material used to assess it and investigate its cognitive foundations. Traditionally, EI has been conceptualized by two theoretical approaches: mixed models and ability models. According to the latter, EI is defined as “the ability to monitor one’s own and others’ feelings and emotions, to discriminate among them, and to use this information to guide one’s thinking and actions”^18^, p. 189. Because it concerns information that has personal relevance and potentially impacts the emotions, EI is called ‘hot intelligence’. Despite its ‘hot’ nature, EI is still ‘intelligence’, which is a cognitive ability correlated with general intelligence (*g*) test scores, according to the positive manifold rule^19^. This relationship has been repeatedly demonstrated when EI has been assessed with performance-based ability EI tests; however, self-report measures have rarely shown similar correlations^20,21^. As ‘intelligence’, EI should be supported by the same cognitive processes that support general abilities. Although some previous studies have examined these relationships, their results are inconclusive. The reason might be that different cognitive processes (attention, working memory, decision making, etc.) have often been analyzed together, although each of them might be related to EI in a different manner. The solution to this problem should lie in analyzing each of the cognitive processes separately; therefore, we decided to focus on working memory.

Some preliminary evidence concerning the EI-WMC relationship was found by Gutiérrez-Cobo, Cabello, and Fernández-Berrocal^21^, who attempted to determine whether the relationship between EI and WMC was dependent on the emotional content (‘hot’ or ‘cool’) of tasks involving WM. Using n-back tasks, the authors provided evidence for the better performance of higher-EI participants (specifically in the managing branch of EI) measured through a performance-based ability test, but only in a hot WMC task with emotional content (both positive and negative).

In this paper, we continue investigating the EI-WM relationship, concentrating on a crucial executive function, i.e., working memory updating (WMU). WMU is known to be one of the three executive functions that reflect individual differences in higher cognition^22^; it is also the firmest predictor of fluid intelligence (e.g.,^23–26^). Moreover, WMU has been claimed to be the only executive process that predicts fluid intelligence^27^. It seems to be a crucial predictor of children’s academic achievement^28^, children’s arithmetical performance^29^, development of rule-guided behavior^30^, students’ reading comprehension and academic achievement^31^ and insight problem solving^32^.

If WMU is important for a wide range of higher-order processes, updating of emotional content in WM could be crucial for emotionally intelligent behavior. There is some evidence to support this assumption. For example, Flores and Berenbaum^33^ showed that removing irrelevant negative WM content relates to the regulation of emotion in social contexts. They argued that this could be a one-way process in which supportive social relationships protect against psychological distress. Additionally, the removal of irrelevant WM content is considered to be a specific WMU process^34^. Although Flores and Berenbaum^33^ investigated the impact of the social regulation of emotions on updating negative WM content, they also realized that releasing WM from irrelevant information, which leads to the conservation of cognitive resources, helps keep cognitive resources available for taxing cognitive tasks so that those tasks can be performed more effectively. In our opinion, this effectiveness extends to intrapersonal and interpersonal situations of emergent behavior that can be considered emotionally intelligent.

To test the hypothesis that updating emotional content in WM could be crucial for emotionally intelligent behavior, we constructed a cognitive task that requires the engagement of the WMU process, which is one of the factors responsible for shaping WMC. This task has two parallel versions: one involves emotionally neutral material, and the other uses emotionally meaningful human faces as stimuli. Using the structural equation approach, we sought to demonstrate that *g* is dependent on the efficiency of memory updating for both emotional and neutral stimuli, whereas emotional intelligence might depend only on the efficiency of WMU in a specific emotional context.

## Method

### Participants

A total of 123 women participated in the first part of the study (age: *M* = 39.71, *SD* = 8.67 years), during which all the measures were administered with the exception of the memory updating task (MUT). The MUT measurements were performed in a separate meeting. Only a subset of the total group (*n* = 69 women) participated in the second part of the study. Informed consent was obtained from all individual participants in the study.

### Measures

#### Fluid intelligence

Raven’s Advanced Progressive Matrices (RAPM)^35^ served as a tool to assess gF.

#### Crystallized intelligence

Verbal intelligence was measured using a test based on Horn and Cattell’s theory of crystallized intelligence (gC). The test requires the classification of as many words as possible into one of five categories (art, biology, science, literature, or geography/history) within 5 min. The test contains 120 such words. The gC indicator is the sum of the correct answers.

#### Emotional intelligence

Emotional intelligence was assessed with the TIE^36^, an ability test based on the EI model developed by Mayer and Salovey^37^. The authors split EI into four ‘branches’: (1) perception, (2) facilitation, (3) understanding, and (4) managing emotions. The TIE consists of two separate parts: in the first part, which relates to the perception and understanding subscales, the participants are asked to identify the feelings and thoughts of individuals involved in the described situations; in the second part, which relates to the facilitation and management subscales, the participants are asked to indicate the most favorable action that an individual should apply to deal with a given situation.

The test contains 24 tasks (6 for each of the four subscales). In each of them, the participants receive a description of a problem situation followed by three alternative responses. The participant’s task is to score each response on a 5-point Likert scale.

Emotional quotient is calculated from the similarity of the participants’ responses to the answers of competent judges (52 experts in psychology, i.e., licensed and experienced therapists, trainers, and HR managers). The obtained points are summed separately for the individual branches and for the whole test (emotional quotient). The overall TIE reliability is *r* = 0.88. The TIE score correlated with RAPM at the level of r = 0.35, *p* < 0.001 and with the Horn test at the level of *r* = 0.26, *p* < 0.001^35^.

#### MUT (memory updating task)

**Figure 1:**
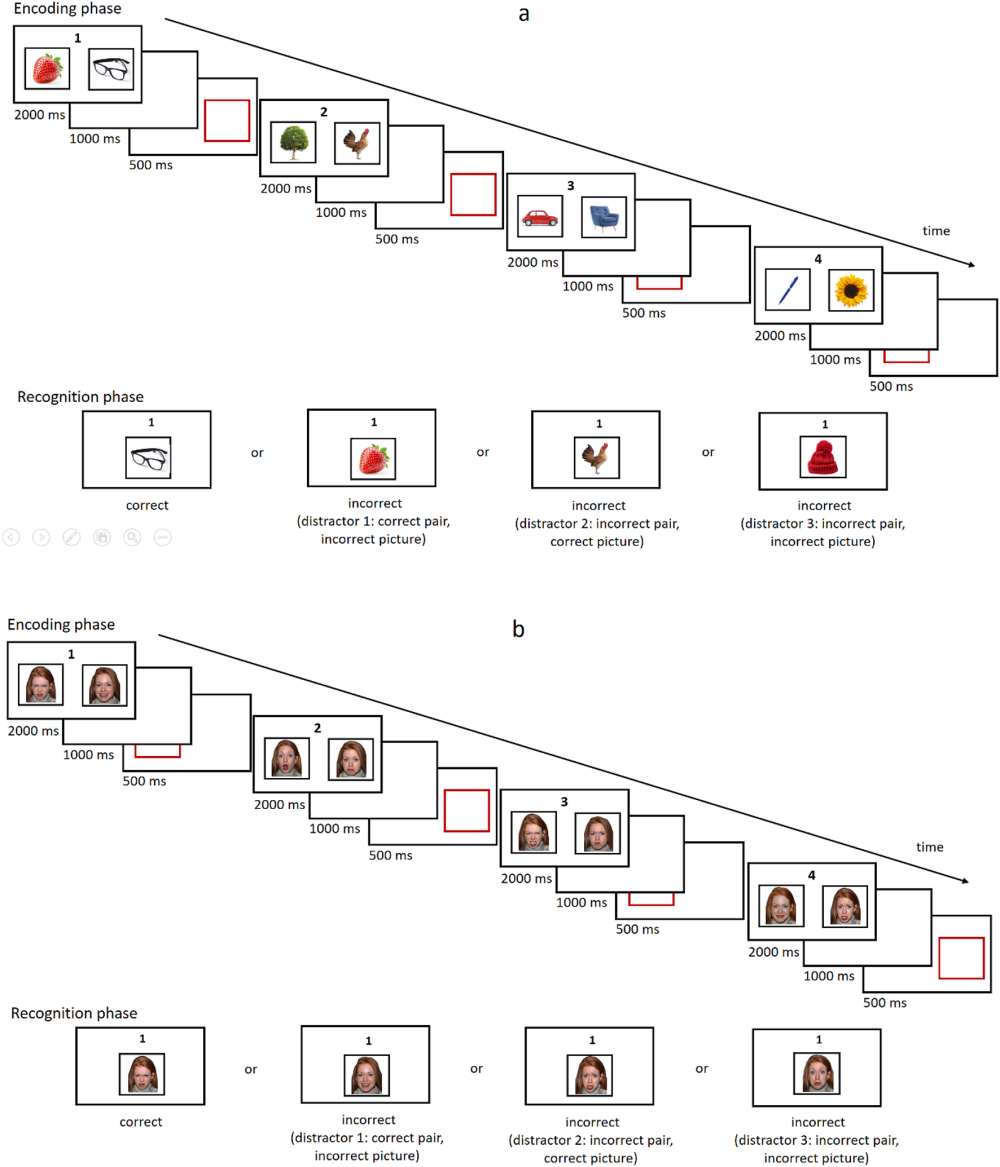
Presentation sequences for the MUT neutral version (**1a**) and MUT emotional version (**1b**) with examples of stimuli. Neutral pictures were taken from free stock pictures, https://www.freepik.com. Emotional pictures were taken from the NimStim Set of Facial Expression^38^, http://www.macbrain.org/resources.htm.

An original computer task, the MUT, was used as a measure of the efficiency of WMU. During this task, the participants see a pair of pictures on a computer screen. In the neutral content condition, the photos show common objects (e.g., a chair or a car); in the emotional content condition, the photos show faces expressing emotions. Each pair of pictures is exhibited for 2000 ms; after an interval of 1000 ms (blank screen), a red frame appears for 500 ms in place of one of the photos. The red frame indicates to the participant which picture should be remembered. Each trial consists of four pairs of photos presented sequentially (a fixed set size) (see Fig. 1).

The participants’ task is to remember which element was indicated in each pair; therefore, they need to encode both the number of each pair and the indicated element. The second picture in each pair becomes a distractor, but since it is not immediately known which element will be a target and which will be a distractor, both elements must initially be remembered. WM updating is possible only after a delayed presentation of the frame. After the sequential presentation of the pictures, in the recognition phase, the participants are shown a pair number from 1 to 4 and a picture. Their task is to decide whether this picture was indicated in the pair with the given number. The participants should answer ‘yes’ only in the case of full accordance between the number of the pair and the picture; in any other case, the answer should be ‘no’.

In addition to the manipulation of content (neutral vs. emotional), the MUT contains two levels of similarity between the exposed objects and three types of distractors. For similarity, the neutral condition uses pictures of objects from different categories, e.g., a chair and a car (low similarity), or from the same category, e.g., two different cars (high similarity). In the emotional condition, the pictures of faces depict different people (low similarity) or the same person (high similarity). The distractors belong to one of three categories: (1) pictures from the correct pair but not indicated by the frame, (2) pictures indicated by the frame but from another pair, or (3) pictures from outside the set that did not appear in a given series at all. The set of emotional pictures was selected from the NimStim Set of Facial Expressions^38^. The set of neutral stimuli was supplemented with free stock pictures from https://www.freepik.com. The two sets were substantially different in the valence dimension, but all the pictures were balanced in terms of physical characteristics.

In summary, the experimental plan of the MUT contains 2 types of content (neutral vs. emotional) × 2 levels of similarity (low vs. high) × 4 types of responses (positive vs. ‘not indicated’ vs. ‘from another pair’ vs. ‘from outside the set’). There are 96 trials in the task, distributed evenly across all conditions. All the participants are subjected to the experimental manipulation, which consists of two separate blocks: neutral and emotional. The rest of the conditions are randomized within the block. The accuracy of the responses and the reaction times are recorded during the task.

### Procedure

All the participants first completed a battery of paper-and-pencil tests in the following order: RAPM, Horn and Cattell’s test, and TIE. The time limit for the RAPM was 40 min, 5 min were allowed for Horn and Cattell’s test, and 30 min were allowed for the TIE. At the second meeting, the participants performed the neutral and emotional versions of the MUT. The counterbalancing was used with a randomly chosen order of the performed versions of the MUT. The research session took approximately 45 min.

### Statistical analysis

To test the relations among the critical constructs in our study, structural equation modeling (SEM) with maximum likelihood estimation was conducted using AMOS 21.0 software^39^. We used full information maximum likelihood (FIML)^40^ estimation as a method of dealing with missing data. The goodness of fit of the model was tested with the χ^2^ test (a nonsignificant test result indicates a good fit), comparative fit index (a CFI value greater than 0.95 indicates a good fit), root mean square error of approximation (RMSEA < 0.06), and standardized root mean square residual (SRMR < 0.08)^41^. As an indicator of the efficiency of WMU in our analysis, we used the percentage of correct answers given by the participants separately for both versions of the MUT (for example, a score of 0.60 indicates that 60% of a participant’s answers were correct).

### Ethical approval

All procedures performed in studies involving human participants were in accordance with the 1964 Helsinki Declaration and its later amendments or comparable ethical standards. The study was approved by the committee assigned by SWPS University of Social Sciences and Humanities.

## Results

Table 1 shows the zero-order bivariate correlations (Pearson’s *r*) between measures of IQ (Horn and RAPM test scores), measures of EI (TIE overall score and subscale scores) and indicators of WMU efficiency (MUT neutral and emotional versions), along with basic descriptive statistics for these variables. Our analysis revealed that the TIE scores in our female sample were more strongly related to the results of the MUT that used emotional stimuli than to the one that used neutral stimuli. Additionally, the test scores for both measures of IQ, i.e., the RAPM test and the Horn test, seemed to be positively related to both versions of the MUT. This result is consistent with the available literature^21^ and shows that performance on some cognitive tasks (e.g., through WMC) may be related to measures of *g* on a very basic level and may not depend on the type of stimuli used in the task (both versions of the MUT required the same basic type of operations using different types of stimuli).

**Table 1:** Descriptive statistics and correlation coefficients for all variables included in the analysis.

Variable name	N	Mean	SD	1	2	3	4	5	6	7	8	9
1. TIE: general score	123	25.64	6.24	1	0.84**	0.87**	0.84**	0.76**	0.28*	0.53**	0.23	0.47**
2. TIE: perception	123	7.12	2.33		1	0.67**	0.55**	0.46**	0.26*	0.43**	0.22	0.31*
3. TIE: understanding	123	6.02	1.84			1	0.66**	0.53**	0.21*	0.45**	0.21	0.46**
4. TIE: facilitation	123	6.41	1.73				1	0.62**	0.28**	0.48**	0.17	0.41**
5. TIE: management	123	6.09	1.62					1	0.15	0.37**	0.13	0.41**
6. RAPM	121	12.43	5.49						1	0.46**	0.22	0.33*
7. Horn	120	36.01	18.98							1	0.42**	0.37*
8. MUT neutral	69	0.61	0.13								1	0.48**
9. MUT emotional	68	0.57	0.09									1

**p < 0.001; *p < 0.05.

To further investigate how both MUT versions relate to general intelligence and emotional intelligence, we moved beyond simple bivariate methods and created a structural equation model. This approach was better suited to our hypothesis, as it does not rely on fragmentary correlations between two variables but allows the creation of latent factors and the testing of multiple relationships within one model (e.g.,^42^).

**Figure 2:**
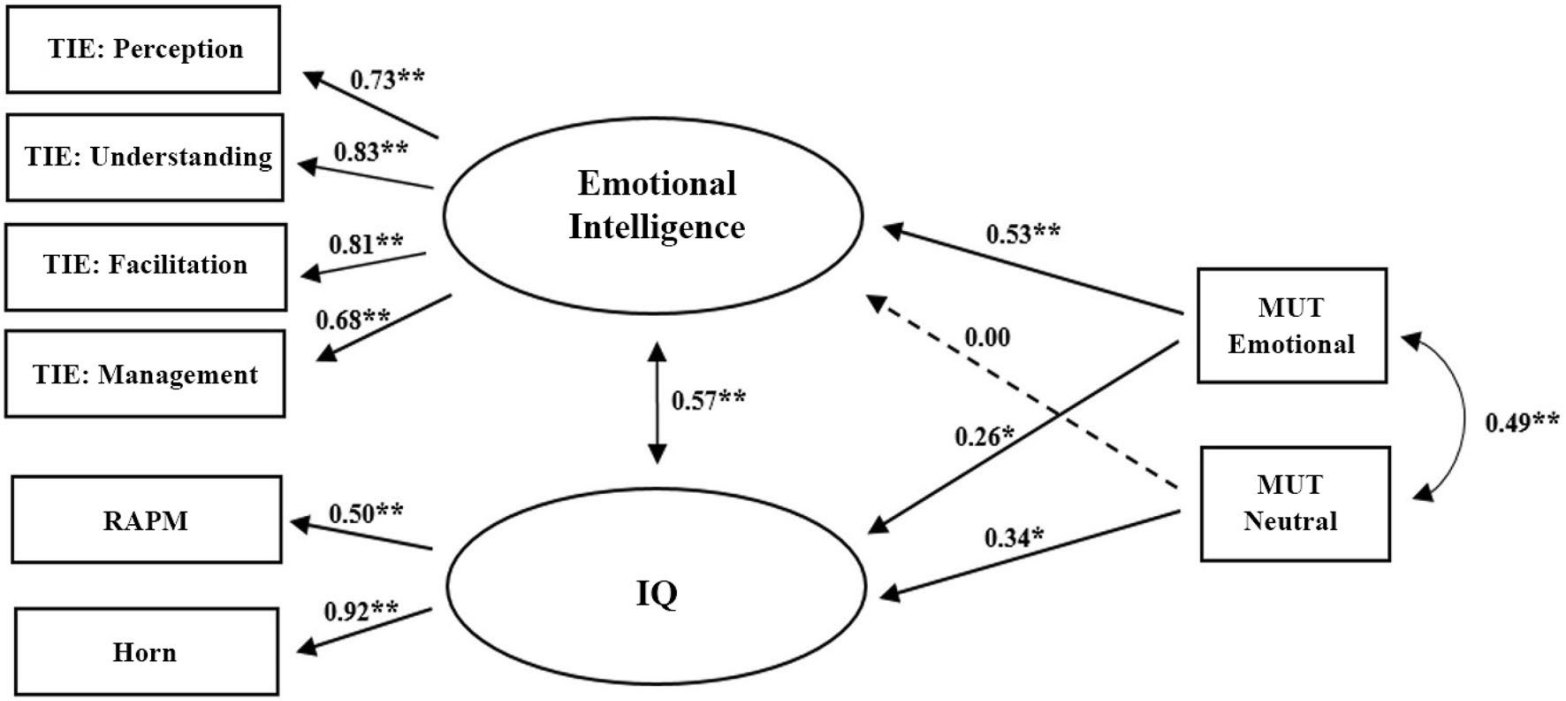
**p < 0.001; *p < 0.05. Structural equation model depicting the pattern of relations between two latent factors of EI and IQ and efficacy of memory updating based on emotional or neutral stimuli (MUT emotional and MUT neutral, respectively). The model shows standardized path coefficients. All coefficients for paths denoted with continuous lines were significant, and these paths were included in the final version of our model. The relationship denoted with the dashed arrow was not significant and was not included in the final version of our model.

In the analyzed model, we tested the relations between the efficacy of WMU based on emotional (MUT emotional) and cognitive (MUT neutral) processes and two latent factors that represent EI and IQ (Fig. 2). We predicted that the emotional version of the MUT would be more strongly related to EI, whereas the version of the task based on neutral stimuli would be more strongly related to IQ. This distinction would reflect different, distinguishable types of WMU processes for neutral and emotional stimuli that could constitute a basis of IQ and EI, respectively. The described relations are depicted in Fig. 2. First, based on our female sample, we tested the measurement model, which consisted of two latent factors (EI and IQ) and their indicators as well as the correlation between the factors. All standardized coefficients that reflect the relationships between the TIE subscales and the latent factor of EI and the paths between the latent factor of IQ and both the RAPM and Horn scores were highly significant (*p* < 0.001) and moderate to high in power (standardized regression coefficients between *β* = 0.50 and *β* = 0.92). The measurement model was fairly well fitted: *χ*^*2*^_8_ = 12.06, *p* = 0.149; CFI = 0.985; RMSEA = 0.064.

In the second step, we introduced two versions of the MUT as predictors of the EI and IQ latent factors (Fig. 2). In relation to our predictions, the version of the MUT based on neutral stimuli was related to the latent factor of IQ (*β* = 0.34, *p* = 0.008) but not to that of EI (*β* = 0.003, *p* = 0.980). Correspondingly, the participants’ scores obtained in the version of the MUT based on emotional stimuli were significantly related to both latent factors: EI (*β* = 0.53, *p* < 0.001) and IQ (*β* = 0.26, *p* = 0.044). However, it is worth noting that the first of the mentioned relationships is exactly twice as strong as the second one. This pattern of results confirms our predictions of the pattern of relations between memory updating in different domains and distinct types of intelligence.

Additionally, our analysis revealed a highly positive covariance between the two types of intelligence tested within the model (*r* = 0.57, *p* < 0.001). As both indicators of WMU in our model were based on the same type of task, we added the covariance between the residual error terms of these variables (*r* = 0.49, *p* < 0.001).

In the next step, we created a constrained version of our model in which an nonsignificant path between the MUT neutral and the latent factor of EI was constrained to 0. Then, we checked whether the unconstrained and constrained versions of our model differed significantly in terms of goodness of fit. The tested difference was not significant, *χ*^*2*^_1_ = 0.01, *p* = 0.980, which indicated that the constrained paths did not bring any additional informative value to the model^41^. Therefore, we excluded the nonsignificant path (depicted by the dashed arrow in Fig. 2) from the discussed model. The final version of our model, which included all the continuous arrows depicted in Fig. 2, was fairly well fitted: *χ*^*2*^_16_ = 19.93, *p* = 0.223; CFI = 0.990; RMSEA = 0.038.

## Discussion

The primary aim of our study was to investigate the WM basis of two types of intelligence: general and emotional. To achieve this aim, we examined two types of WMU tasks based on neutral or emotional stimuli. Using a structural equation model, we analyzed the relationships between latent factors of IQ and EI on the one hand and both types of WMU efficacy on the other hand. Our results show that the two types of intelligence are positively related: relationships between the TIE global score and both gC (Horn test) and gF (RAPM) indicators were positive and significant (*r* = 0.53 and *r* = 0.28, respectively). This result for a female sample is in line with previous research based on participants of both genders and shows that EI ability tests tap into both components of *g*: crystallized (acculturated knowledge, heavily dependent on education and experience) and fluid (innate ability, not heavily dependent on acquired knowledge). However, the gC component is usually stronger^43^. Therefore, the correlation between EI tests and measures of gC seems to be stronger than with gF measures^44–46^, which is also the case in our study.

The standardized estimate of the relationship between the two latent factors in our model was also positive and highly significant (*β* = 0.57, *p* < 0.001). This result is consistent with previous studies that showed a positive relationship between measures of IQ and EI when the latter is measured using ability tests (as it was in our study) but not when it is assessed by questionnaires based on self-assessments^20,21^ or appraised by an external observer^47^.

Concerning the main research question, our analysis revealed that for females, the efficacy of WMU for neutral stimuli is positively related to IQ (*β* = 0.34), but the predictive power of this type of WMU vanishes for EI (*β* = 0.003, *p* = 0.980.). On the other hand, among females, the efficacy of memory updating based on emotional stimuli significantly predicts not only IQ (*β* = 0.26, *p* = 0.044) but also EI (*β* = 0.53, *p* < 0.001). It is worth noting that the latter relationship is stronger than the former, which indicates that WMU based on emotional stimuli constitutes a firmer basis for EI than for IQ.

How do these results fit into the landscape of previous studies? The relationship between WM and IQ seems to be well understood, and our results provide further evidence for a strong role of WMC in shaping cognitive abilities^3,4,9^. The latent factor of IQ significantly relates to both types of WMU, so this relationship seems to be nonspecific and not heavily dependent on second-order conditions such as context or the type of stimuli temporarily stored in WM. What is new is that our results indicate that the relationship between WMU and EI is conditional: it is significant only when WM is operating on emotional content, i.e., material that is directly relevant to EI itself. In other words, the relationship between EI and WM seems to be much more domain specific than that between IQ and WM. Females with high EI, compared to those with low levels of emotional abilities, are significantly more effective in memory updating when a task is based on emotional stimuli. Such efficiency probably facilitates further processing of affective stimuli by assimilating and managing only relevant information and not negligible noise.

Our study, although based solely on a female sample, takes a step forward in understanding the cognitive underpinnings of EI. Considering that only the aforementioned individual studies^20,21^ investigated the relationship between WM and EI, the results we obtained are relatively novel and important.

### Limitations

Our results should be considered with caution because of the relatively small sample size (*n* = 123 for measures of EI and IQ and *n* = 69 for the MUT) and its characteristics (only women). The conclusions described above can be safely extrapolated only to females. To generalize the obtained pattern of relationships, future research should also investigate the relationship between EI and WMU for males. Although the sample size is a constraint for the present study, the number of variables in our model is also small (8 observed indicators); therefore, the model is only slightly below the threshold of 10 participants per indicator, which is often cited as a rule of thumb for sample size in SEM^48^. Moreover, most factor loadings for indicators of EI and IQ are relatively high. Last, the skewness and kurtosis of most indicators used in the model have acceptable values (skewness within the value of |1| is observed for all variables and kurtosis within the value of |1.2| for 7 of the 8 variables). There is evidence that high factor loadings and normal distribution of data decrease the minimum sample size required for SEM^49,50^. One additional issue that should be noted is that further studies should replicate our findings using different measures of IQ, EI and WMU to confirm that our results are not measure-specific and are universal across various methodological approaches.

## References

[1] Symonds PM, Spearman C (1928). The abilities of man; their nature and measurement. J. Philos..

[2] Valentin Kvist A, Gustafsson JE (2008). The relation between fluid intelligence and the general factor as a function of cultural background: a test of Cattell’s Investment theory. Intelligence.

[3] Chuderski A, Necka E (2012). The contribution of working memory to fluid reasoning: capacity, control, or both?. J. Exp. Psychol. Learn. Mem. Cogn..

[4] Colom R, Rebollo I, Palacios A, Juan-Espinosa M, Kyllonen PC (2004). Working memory is (almost) perfectly predicted by g. Intelligence.

[5] Kane MJ, Hambrick DZ, Conway ARA (2005). Working memory capacity and fluid intelligence are strongly related constructs: comment on Ackerman, Beier, and Boyle (2005). Psychol. Bull..

[6] Kyllonen PC, Christal RE (1990). Reasoning ability is (little more than) working-memory capacity?!. Intelligence.

[7] Nȩcka E (1992). Cognitive analysis of intelligence: the significance of working memory processes. Pers. Individ. Differ..

[8] Oberauer K, Wilhelm O, Schulze R, Süß HM (2005). Working memory and intelligence—their correlation and their relation: comment on Ackerman, Beier, and Boyle (2005). Psychol. Bull..

[9] Oberauer, K., Süß, H. M., Wilhelm, O. & Sander, N. Individual differences in working memory capacity and reasoning ability. In *Variation in Working Memory* (Oxford University Press, 2012).

[10] Daneman M, Carpenter P (1980). Individual differences in working memory and reading. J. Verbal Learn. Verbal Behav..

[11] Henry LA, Messer DJ, Nash G (2014). Testing for near and far transfer effects with a short, face-to-face adaptive working memory training intervention in typical children. Infant Child Dev..

[12] Daneman M, Carpenter P (1980). individual differences in working memory and reading. J. Verbal Learn. Verbal Behav..

[13] Daneman M, Merikle PM (1996). Working memory and language comprehension: a meta-analysis. Psychon. Bull. Rev..

[14] De Smedt B (2009). Working memory and individual differences in mathematics achievement: a longitudinal study from first grade to second grade. J. Exp. Child Psychol..

[15] Nęcka, E. & Orzechowski, J. Higher-order cognition and intelligence. in *Cognition and Intelligence: Identifying the Mechanisms of the Mind* 122–141 (Cambridge University Press, 2004).

[16] Süß HM, Oberauer K, Wittmann WW, Wilhelm O, Schulze R (2002). Working-memory capacity explains reasoning ability—and a little bit more. Intelligence.

[17] Gottfredson LS (1997). Why g matters: the complexity of everyday life. Intelligence.

[18] Salovey P, Mayer JD (1990). Emotional intelligence. Imagin. Cogn. Pers..

[19] Mayer, J. D., Caruso, D. R. & Salovey, P. Selecting a measure of emotional intelligence. The case for ability scales. in *The Handbook of Emotional Intelligence: Theory, Development, Assessment, and Application at Home, School, and in the Workplace* 320–342 (2000).

[20] Gutiérrez-Cobo MJ, Cabello R, Fernández-Berrocal P (2016). The relationship between emotional intelligence and cool and hot cognitive processes: a systematic review. Front. Behav. Neurosci..

[21] Gutiérrez-Cobo MJ, Cabello R, Fernández-Berrocal P (2017). The three models of emotional intelligence and performance in a hot and cool go/no-go task in undergraduate students. Front. Behav. Neurosci..

[22] Miyake A (2000). The unity and diversity of executive functions and their contributions to complex ‘frontal lobe’ tasks: a latent variable analysis. Cogn. Psychol..

[23] Friedman NP (2006). Not all executive functions are related to intelligence. Psychol. Sci..

[24] Benedek M, Jauk E, Sommer M, Arendasy M, Neubauer AC (2014). Intelligence, creativity, and cognitive control: the common and differential involvement of executive functions in intelligence and creativity. Intelligence.

[25] Wongupparaj P, Kumari V, Morris RG (2015). The relation between a multicomponent working memory and intelligence: the roles of central executive and short-term storage functions. Intelligence.

[26] Ren X, Schweizer K, Wang T, Chu P, Gong Q (2017). On the relationship between executive functions of working memory and components derived from fluid intelligence measures. Acta Psychol. (Amst).

[27] Chen T, Li D (2007). The roles of working memory updating and processing speed in mediating age-related differences in fluid intelligence. Aging Neuropsychol. Cogn..

[28] Lechuga MT, Pelegrina S, Pelaez JL, Martin-Puga ME, Justicia MJ (2016). Working memory updating as a predictor of academic attainment. Educ. Psychol..

[29] Iuculano T, Moro R, Butterworth B (2011). Updating Working Memory and arithmetical attainment in school. Learn. Individ. Differ..

[30] Amso D, Haas S, McShane L, Badre D (2014). Working memory updating and the development of rule-guided behaviour. Cognition.

[31] García-Madruga JA, Gómez-Veiga I, Vila J (2016). Executive functions and the improvement of thinking abilities: the intervention in reading comprehension. Front. Psychol..

[32] Nęcka E, Żak P, Gruszka A (2016). Insightful imagery is related to working memory updating. Front. Psychol..

[33] Flores LE, Berenbaum H (2017). The social regulation of emotion and updating negative contents of working memory. Emotion.

[34] Ecker UKH, Lewandowsky S, Oberauer K (2014). Removal of information from working memory: a specific updating process. J. Mem. Lang..

[35] Raven, J. & Raven, J. Raven Progressive Matrices. in *Handbook of Nonverbal Assessment* 223–237 (Springer, 2003).

[36] Śmieja M, Orzechowski J, Stolarski MS (2014). TIE: an ability test of emotional intelligence. PLoS ONE.

[37] Mayer, J. J., Salovey, P. & Caruso, D. Mayer–Salovey–Caruso Emotional Intelligence Test (MSCEIT). (Multi-Health Systems, Toronto, Canada, 2002).

[38] Tottenham N (2009). The NimStim set of facial expressions: judgments from untrained research participants. Psychiatry Res..

[39] Arbuckle, J. L. *IBM SPSS AMOS 22 User Guide*. *IBM Corps* (Amos Development Corporation, 2013).

[40] Allison PD (2003). Missing data techniques for structural equation modeling. J. Abnorm. Psychol..

[41] Hu LT, Bentler PM (1999). Cutoff criteria for fit indexes in covariance structure analysis: conventional criteria versus new alternatives. Struct. Equ. Model..

[42] Byrne BM (2013). Structural Equation Modeling with AMOS: Basic Concepts, Applications, and Programming.

[43] Webb CA (2013). Convergent and divergent validity of integrative versus mixed model measures of emotional intelligence. Intelligence.

[44] Farrelly D, Austin EJ (2007). Ability EI as an intelligence? Associations of the MSCEIT with performance on emotion processing and social tasks and with cognitive ability. Cogn. Emot..

[45] Mayer JD, Roberts RD, Barsade SG (2008). Human abilities: emotional intelligence. Annu. Rev. Psychol..

[46] Checa P, Fernández-Berrocal P (2015). The role of intelligence quotient and emotional intelligence in cognitive control processes. Front. Psychol..

[47] Boyatzis RE, Batista-Foguet JM, Fernández-i-Marín X, Truninger M (2015). EI competencies as a related but different characteristic than intelligence. Front. Psychol..

[48] Nunnally JC (1967). Psychometric Theory.

[49] Wolf EJ, Harrington KM, Clark SL, Miller MW (2013). Sample size requirements for structural equation models: an evaluation of power, bias, and solution propriety. Educ. Psychol. Meas..

[50] Kline RB (2011). Principles and Practice of Structural Equation Modelling.

